# Mental health and spiritual well-being in humanitarian crises: the role of faith communities providing spiritual and psychosocial support during the COVID-19 pandemic

**DOI:** 10.1186/s41018-022-00127-w

**Published:** 2022-10-18

**Authors:** Ellen Goodwin, Kathryn Kraft

**Affiliations:** 1 SOAS, London, UK; 2 University of East London, London, UK

**Keywords:** Psychosocial, Spiritual, Religion, Faith, Mental health, Faith leaders, Humanitarian, COVID-19

## Abstract

Across the globe, people’s reactions to the COVID-19 pandemic and its accompanying lockdowns highlighted, and continued to stress, the relevance of mental health and psychosocial support (MHPSS) in responding to crises, including in low-income and emergency settings. They also demonstrated the importance of faith practices and communities of faith as core needs and important coping mechanisms for many affected people in times of crisis. Drawing on data collected by World Vision International, the world’s largest child-focused humanitarian and development organisation, in the course of its response to COVID-19 in 70 different countries, this article explores the ways in which faith groups and faith leaders responded to the perceived needs of their communities. While keen to offer psychosocial support to people suffering anxiety and fear in the context of the crisis, this has often been integrated with spiritual support. Analysing their work from the framework of a rights-based approach to mental health, we conclude that they did contribute to psychosocial support through their MHPSS, in part through their provision of spiritual care. However, spiritual well-being should not be conflated with psychosocial well-being. While faith groups play an important role in MHPSS, their primary role is to offer spiritual care.

## Introduction

Faith remains extremely important in the lives of people all over the world, and, counter to earlier presumptions, it is becoming more so. Research indicates that, globally, eight-in-ten people — and rising — identify with a faith group (Hackett et al. 2012, p.9). Faith plays an integral role in how many people understand their place in the world and the decisions they make for themselves and their communities (Jones and Peterson 2011:1296; Ammerman 2020). Faith is therefore inextricably linked to the mental health and psychosocial well-being of individuals and communities all over the world (Schafer 2010).

People’s reactions to the COVID-19 pandemic across the globe, with its accompanying lockdowns, highlighted, and continued to stress, the relevance of mental health and psychosocial support (MHPSS) in responding to crises, including in low-income and emergency settings. Even in the early days of the pandemic, it was clear that COVID-19 would have direct and indirect psychological and social impacts on people around the world, affecting their mental health (Holmes et al. 2020, p.547). The extraordinary global situation resulting from the rapid spread of the COVID-19 virus saw symptoms of anxiety and stress increase, as did the risk that already clinically relevant people would be more likely to engage in harmful behaviours. The secondary impacts of the pandemic, including the rampant economic fall-out, increased the likelihood of these symptoms. The isolation imposed on individuals as a result of national management policies had the potential to increase feelings of loneliness which are themselves strongly associated with ‘anxiety, depression, self-harm, and suicide attempts across the lifespan’ (Holmes et al. 2020, p.548). In response, Holmes et al. (2020, p.547) called on research funders and researchers to make the effort to better understand the impacts of COVID-19 on mental health and psychosocial well-being.

While Holmes et al.’s (2020) work is focused on the high-income context of the UK, there is evidence that the mental health and social impacts of COVID-19 were just as, if not more, significant in humanitarian contexts. Even before the COVID-19 pandemic, it was estimated that around one in five people living in conflict zones has some sort of mental illness, two times higher than the general population (OCHA 2020). An increased demand posed a challenge for the humanitarian sector as MHPSS services in these contexts are often limited or non-existent. Additionally, the services that are available faced disruption as a result of the COVID-19 pandemic (Reliefweb 2020).

As the COVID-19 pandemic progressed, concerns around its impact on mental health and psychosocial well-being became a reality. For example, in north-western Syria, between April and May 2020, double the amount of people received consultations for their mental health in comparison with the same time the year before (OCHA 2020). Haddad et al. (2020) have called the mental health and psychosocial struggles associated with COVID-19 the ‘silent pandemic’. Haddad et al. (2020) focus on the impacts of the pandemic on the mental health and well-being of children in conflict-affected situations, stressing their concern that children in these contexts were struggling to cope with the impacts of the COVID-19 pandemic. The pandemic compounded the ‘fear, trauma and chronic stress’ already faced by those living in conflict-affected situations (Haddad et al. 2020, p.4). According to Haddad et al.’s report (Haddad et al. 2020, p.4), more than half of children living in fragile and conflict-affected situations expressed the need for MHPSS as a result of COVID-19.

The ‘silent pandemic’ speaks to the deterioration of mental health and psychosocial well-being as a result of the COVID-19 pandemic, but it has also highlighted the importance of faith and faith communities as important coping mechanisms in times of crisis. Many struggling with COVID-19 and its secondary impacts found that their faith and membership of a faith community were an important resource in their ability to cope (see Blair 2020; Miller 2020). The COVID-19 pandemic reaffirmed a role for faith in fostering positive and negative meaning-making processes, potentially promoting and potentially hindering individual and community resilience. Either way, faith and communities of faith are inextricably linked to people’s experiences of crises and their ability to cope and recover. This paper will explore how faith and communities of faith contributed to mental health and psychosocial well-being during the COVID-19 pandemic through a rights-based lens.

Furthermore, while there is increasing engagement in the academic literature with the roles of faith for promoting a secular framing of MHPSS, such as a rights-based approach, there remains a lack of engagement with faith as an end in itself for supportive care. Discussions around the roles of faith for MHPSS continue to overlook or even avoid engaging with spiritual support as an end in itself, which may be a potentially significant resource for communities of faith in times of crisis. In order to help fill this gap in understanding within the academic literature and humanitarian communities of practice, this paper will also explore how, during the COVID-19 pandemic, spiritual support was seen as a core need by many. During the COVID-19 pandemic, as in many other crises, many sought spiritual support from faith actors, delivered as part of their wider support work with their congregations. We will argue that while faith-based care may be an important contributor to MHPSS, faith is itself a deeply felt core need of many affected people, beyond the objectives of MHPSS. This leads us to reflect on how the experience of the International Faith-Inspired Organisation (IFIO) World Vision highlights the importance of spiritual support and spiritual well-being as an objective which may surpass the importance of mental health and psychosocial well-being, offering an important reminder that while faith groups play an important role in MHPSS, their primary role is to offer spiritual care.

## Methodology

This paper draws on existing research and evidence, as well as primary data gleaned from a number of sources of data collected in collaboration with World Vision International. World Vision is a global Christian child-focused organisation delivering development, humanitarian and advocacy work in approximately 100 countries around the world. When the COVID-19 crisis erupted, World Vision quickly pivoted a significant portion of its development funding to respond to the effects of the pandemic and resultant lockdowns in 70 different countries, to serve upwards of 51 million people including 22 million children, within in the first 6 months after the pandemic was declared. World Vision’s response included health interventions, programmes to support children’s holistic well-being and other community-based activities. Much of this programming capitalised on existing relationships with local faith groups, both Christian and of other faith traditions, to share messages about safe hygiene practices and health updates and combat misinformation as well as to support faith leaders’ desire to support the emerging varied needs of their own congregations and communities. In 2020, World Vision collaborated with a reported 206,000 faith leaders around the world to address the fall-out of the pandemic. In an internal survey of 317 faith leader partners, World Vision found that all of them were offering some form of psychosocial support to their communities during the early months of the pandemic.

World Vision asked each field office to submit biweekly updates (called ‘sitreps’) on their COVID-19 programming and in each update asked to include a narrative of what they did with faith partners. A total of 48 countries reported on their work with faith groups. There were various different ways in which different World Vision teams may have engaged with these groups, according to their understanding of people’s needs and priorities in their context, and so they were given a fair bit of leeway in how they reported about their work. Some reported information sharing, others training or material support, others networking and others joint advocacy. Some reported numbers of participants in specific activities, while others reported only the total number of faith actors with whom they worked.

We were given access to these reports from March through August 2020, and it is the content of this narrative reporting which provides the main source of data upon which the analysis in this paper is based. It is important to note that this data is project monitoring data and was not collected for research so has not gone through an ethics review board. Furthermore, we were given permission to use the data for our analysis but were asked not to use direct quotes or name specific countries. We therefore conducted a thematic analysis of the narrative reports to identify key issues, areas of learning and commonalities in engagement emerging across different contexts. Fully recognising the diversity of communities, countries and regions which this data represents, the data is assessed for common themes in the light of a crisis which is uniquely global and which has affected communities around the world in overlapping ways.

Based on our review of World Vision’s emerging data on its faith partnerships to respond to COVID-19, we make the case that while spiritual well-being and mental health are by no means the same thing, the responses of communities of faith to crises like COVID-19 often integrate MHPSS through a holistic approach in which their priority is to meet the spiritual needs of their congregants.

## Background

The humanitarian arena is going through a process of global reform which was encapsulated at the first-ever World Humanitarian Summit (WHS) in 2016 (Aneja 2016). The summit called on states and other relevant stakeholders to commit to 5 responsibilities: prevent and end conflict, respect the rules of war, leave no one behind, work differently to end need and invest in humanity. It is in this context of humanitarian reform that the humanitarian sphere has turned its attention to the importance of mental health and psychosocial well-being. The WHS did not highlight the importance of MHPSS at the formal level; however, MHPSS is relevant across the themes and priorities explored at the WHS. As a result, MHPSS was a frequent subject of conversation at and around the summit (Galappatti 2016).

MHPSS has become an increasing focus of the humanitarian sphere since the WHS as it speaks to the desire to reduce risk, vulnerability and overall need. The provision of MHPSS looks not just to respond to the immediate needs of individuals and communities after crises but also to help them deal with and respond to increasingly frequent, protracted and complicated crises in the long term. Part of this long-term plan is the promotion of individual and community resilience. The concept of resilience refers to the ability of systems to ‘…absorb disturbance and recognise while undergoing change so as to still retain essentially the same function, structure, identity and feedback’ (Folke 2006, p.259). MHPSS is increasingly seen as an important part of promoting individual and community resilience. As a result, over the last decade, there has been an increasing number of MHPSS programmes, as well as the publication of the *IASC Guidelines on Mental Health and Psychosocial Support in Emergency Settings*, which seek to help communities recover from, or manage to some extent, the effects of crises.

MHPSS is a descriptor for humanitarian interventions which are intended to preserve or help restore mental health during or in the aftermath of a crisis or traumatic event. According to the World Health Organization, ‘Mental health is a state of well-being in which an individual realises his or her own abilities, can cope with the normal stresses of life, can work productively and is able to make a contribution to his or her community’ (WHO 2022). This draws a link between medical health, gainful employment and economic activity, social connections and support and freedom from discrimination or social exclusion (Mann et al. 2016, p.264). Much of the literature on mental health care which was developed in stable countries and contexts addresses the same principles and needs as MHPSS, the label which is more often used in humanitarian contexts.

The humanitarian reform agenda which has seen MHPSS emerge as a key focus of humanitarian interventions has led the global community to reflect on what humanitarianism is and who can legitimately be considered humanitarian actors. The shifts being experienced across the humanitarian arena have expanded the space in which different actors, including those not traditionally part of the formal humanitarian sphere, can be involved in the humanitarian arena (see Hilhorst and Jansen 2010). This process of reform and reflection has therefore created the space in which to (re)visit the role of faith in humanitarian processes.

Of course, it is often argued that faith has always been part of humanitarian processes. Some argue that humanitarianism has its roots in faith principles, faith communities and faith-based giving (see Barnett and Stein 2012; Warner 2013). Faith remains central to many communities facing crises, and it is a significant motivation for the majority of humanitarian staff working at the national level (Ager and Ager 2011, p.465). Faith is intertwined with the humanitarian-development-peacebuilding nexus. While evidence suggests that faith is ambivalent towards violence and peace, faith can be both a driver of violence and conflict as well as peace and reconciliation (Gopin 2000; Longman 2001; Philpott 2007; Appleby 2010). Often in failed states, all that is left behind in poor communities, to provide vital services, are faith institutions (Marshall 2004:897).

However, from the nineteenth century until the last few decades, the role of faith was ignored, overlooked and sidelined by the humanitarian sphere. Following the secular codification of humanitarianism after World War II, as well as the rise of neoliberalism and its material bias in the 1980s, not to mention the bureaucratisation, rationalisation and professionalisation of the humanitarian arena over the last two decades, the humanitarian sphere has been reluctant to engage with faith (see Barnett 2012). This distrust is informed by modernisation and secularisation theories which have been dominant across the humanitarian sphere and in the social sciences (Selinger 2004). These theories posit that as countries modernise, faith will become increasingly irrelevant, eventually becoming obsolete all together. Today, such approaches to faith are often viewed as untenable in the age that has seen a failure of modernisation, apparent (re)surgence of faith and renegotiation of humanitarianism. It is also important to note that while in Western Europe modernisation has often been accompanied by radical secularisation, this has not been the case in many other parts of the world where faith never went through processes of privatisation.

Nonetheless, major IFIOs like World Vision have long occupied a prominent role in the humanitarian architecture. IFIOs were part of the extensive global consultations with major humanitarian actors ahead of the WHS, and there is a growing academic literature exploring their potential advantages and challenges for effective humanitarianism (see OCHAi 2016; Gaillard and Texier 2010; Ferris 2011; Ager and Ager 2011; p.457; Tomalin 2015; Occhipinti 2015). Through the nineteenth and twentieth centuries, many IFIOs were incorporated into the humanitarian sphere and subjected to the same secular codification. Indeed, IFIOs emerging in the development/humanitarian sphere at this time tended to adhere to the ‘secular script’ of humanitarianism (Deneulin and Bano 2009; Ager and Ager 2011, p.457; Burchardt 2012, p.31). The World Bank estimates that 50% of health and education services in certain parts of sub-Sharan Africa were provided by FIOs in 2000 (Deneulin and Bano 2009:1; Marshall 2004:897). Meanwhile, the ‘glocal’ nature of many faiths suggests that FIOs can plug into local and global networks, fostering relationships with actors beyond funding based on shared identities, values, beliefs, improving communication and coordination between actors at all levels (Ferris 2011, p.617; Burchardt 2012, p.50; PaRD 2016, p.14).

The work of IFIOs providing MHPSS in emergency settings is a central focus of this paper which is based on data collected by World Vision, a prominent IFIO. The term faith-based organisation (FBO) is more widely used in the academic literature and humanitarian practice. However, FIO is more inclusive of the variety of ways that faith influenced and continues to inspire certain humanitarian organisations. Accordingly, the term FIO is a broad category. It encompasses a wide range of organisations, operating at different levels from international to grassroots organisations, with different relationships to faith (Clarke and Jennings 2008, p.34; Ferris 2011, p.609). The diversity of the category of FIO means it is difficult to make generalisations around the distinctiveness of their work. For this reason, it is useful to focus on the work of specific FIOs, in this case the IFIO World Vision. With its evangelical origins, since the late 1970s, World Vision has described itself as primarily humanitarian, with an interdenominational Christian identity, concerned for both physical and spiritual needs. As it has grown in size and broadened the scope of its programming, its focus has increasingly been on relief and development work (King 2019, p.167).

Bolstered by the emphasis on localisation and collective action at the WHS, there has recently been a shift in focus of humanitarian attention towards local faith actors (LFAs) and local communities of faith (LCFs) and their roles in responding to humanitarian crises. This shift has been felt across the humanitarian arena, including in the work of IFIOs like World Vision who have included faith partnerships in almost all aspects of its COVID-19 response. This is in no doubt due to lessons learnt from previous humanitarian crises, including most notably the Ebola epidemic. During the Ebola epidemic, it became clear that some faith practices such as burial rituals that involved touching highly infectious bodies were contributing to the spread of the disease. While public health specialists tried to work with local communities to change behaviours around burial rites and rituals, it was not until they engaged with local faith and community leaders that they saw communities begin to comply with public health messaging (Featherstone 2015, p.8; PaRD 2016, p.8-9). Local faith leaders were trusted public figures in many communities affected by Ebola, and they were able to modify faith practices in ways that were safe and dignified. Local faith leaders also played a significant role in reducing stigma against Ebola workers as fear of the deadly disease threatened to destroy community cohesion. They used messages of hope based on biomedical knowledge and scripture to respond holistically to the crisis, changing the hearts and minds of their communities (Featherstone 2015, p.9).

While it has taken longer for the humanitarian sphere to engage with LFAs than IFIOs, it is now widely accepted that LFAs tend to be first responders in times of crises (Wisner 2010; Fiddian-Qasmiyeh and Ager 2013). Humanitarian actor’s most common mode of engagement with LFAs is with local faith leaders. It is broadly understood that local faith leaders often have a strong and deep connection with people that is based on a shared faith. Local faith leaders can have large followings and hold a privileged position within their sphere of influence which means they are trusted by their adherents (Aziz 2017, p.1-2). Local faith leaders have a platform, and many nowadays have a significant social media platform, from where their messages can be disseminated quickly and efficiently (Aziz 2017, p.2). As a result, local faith leaders can, and have, played a transformational role in responding to crises (Featherstone 2015, p.8).

Since the pandemic began, in contexts all over the world, LFAs have been at the forefront of responding to COVID-19 and its secondary impacts. Local faith leaders and faith communities possess various resources such as human, financial, social, spiritual and religious capital, enabling them to respond to crises and provide for people’s basic needs in times of crisis (see Verteer 2003; Rivera and Nickels 2014; Baker 2017; Berger and Redding 2017). During the pandemic, local faith institutions and groups have leveraged these resources to provide health care (Gomes 2020; Fraser 2020) and spiritual support (Molina 2020), provide basic services including food (Dhara 2020; Fides 2020) and advocate for and support the most vulnerable (Swiatecki 2020; Allen 2020).

Of course, LFAs, and local faith leaders in particular, also present some challenges for effective humanitarian responses. As was seen during the COVID-19 pandemic, misinformation spread by local faith leaders can lead to problematic behaviours which worsen a crisis. Jones and Peterson (2011) criticise what they perceive to be a narrow focus of local faith leaders, which can disempower other local actors in favour of the most dominant voices within communities of faith. Bradley (2011, p.26-28) expresses concern that working with faith leaders for humanitarian or development purposes risks providing a platform for faith leaders to represent and further promote their essentialist views about women and other marginalised groups.

These valid critiques reinforce the importance of recognising the diversity of the category of faith leaders. In many faith traditions, there are a wide range of formal and informal faith leaders, with their own agency and capacities. While there are formal faith leaders, at the national and local levels, such as ordained faith leaders including priests and Imams, there are also informal faith leaders and lay leaders such as the leaders of prayer groups, youth groups, women’s groups and so on who are particularly prominent at the local level. Whether these various faith leaders presented opportunities and/or challenges for humanitarian responses to crises like COVID-19, the evidence highlights that they are inextricably linked with humanitarian processes. To not engage with relevant faith leaders at all levels leaves their strengths a source of untapped potential and weaknesses as unmitigated obstacles for more effective humanitarian responses.

The literature on LFAs and LCFs, including local faith leaders, for more effective humanitarian interventions, is growing. One of these fledgling areas of research is around the roles of LFAs and LCFs for providing MHPSS in emergency settings. The rise in prominence of concerns about MHPSS within the humanitarian sphere has seen work emerge on the role of faith at the local level, such as the impact of faith narratives, beliefs and practices in fragile contexts (Koening 2007; Peres et al. 2007; Henderson et al. 2010). More of this literature will be explored in the following sections. However, it is important to note that while there is a large literature exploring the intersections between faith and mental health and psychosocial well-being, the literature highlighted above remains predominantly based in western, industrialised contexts, mainly North America and western Europe, with a specific focus on Christian and Jewish beliefs and practice (Walker et al. 2012, p.120). More research building on the work of Ager et al. (2005), Schafer (2010) and Ager et al. (2014), such as this paper, needs to be done to expand understanding of the roles of faith for promoting MHPSS in low-income and emergency settings.

As well as debates around what is a FIO and who constitutes a ‘LFA’, there are more foundational debates around how to define key concepts such as faith. Faith, religion and spirituality, for example, are often used interchangeably. This confluence of faith, religion and spirituality has been criticised for overlooking the variety of diverse traditions and beliefs within a given context (Van den Berg et al. 2011, p.67). It is difficult to create a typology of the concepts of faith, religion and spirituality let alone how they relate to each other, because their individual meanings are contested. It is broadly accepted that religion, spirituality and faith are linked, but distinct, constructs (Schafer 2011, p.74). While there are no agreed definitions of faith, religion and spirituality, there have been numerous attempts to define them and how they relate to each other. Drawing on this body of literature, it is possible to draw out consistent themes from which to construct relevant working definitions.

Religion is often used as a term to denote specific beliefs and practices linked to the supernatural realm and the systems and structures that are created around them (Smith 1995, p.893). The term speaks to institutions and organisational structures. It refers to long-lasting world views and attitudes based on codes of behaviours and symbol systems (Walker et al. 2012, p.116). Religiousness is then often used to refer to individuals and communities expressions of their beliefs (Lunn 2009, p.37). However, as Wilson (2012) argues, religion is not either institutional or ideational, it is not individual or communal and it is not irrational or rational. These binary distinctions imposed upon the definition of religion, which have historically been operational in disciplines like international relations and the social sciences more widely, restrict our understanding of religion’s interactions with other phenomena be it politics or humanitarianism. They have also played a role in sidelining religion in these disciplines. Therefore, while Lunn’s (2009) definition of religion is useful, emphasising the formal aspects of religion and the different hierarchical levels at which religion operates, it is important to also take into account how religion operates at the individual and social level (Haynes 2002, p.17).

Spirituality on the other hand is often used to describe ‘the human search for purpose and meaning of life experiences’ (Sheridan and Amato-von Hemert 1999, p.129) or individuals’ experiences of a transcendent relationship. If religion is about doing, spirituality is about being (Newman 2004, p.106). While the literature has criticised the conflation of religion and spirituality, pointing to their distinctive meanings, it is important in the context of this paper, situated in humanitarian contexts, to point out that this distinction is a relatively recent one. Walker et al. (2012) highlight how this distinction has been produced in western European contexts of reduced church attendance and opposition to organised religion. This distinction between religion and spirituality allows for the assumption that spirituality can exist without religion. However, historically, in western Europe and in many contexts around the world today, this distinction is less salient: the spiritual and physical world are more intertwined, rendering the distinction between religion and spirituality less clear and less significant (Walker et al. 2012, p.117-118).

Spirituality will be used in this paper to refer specifically to ‘spiritual care’ or spiritual well-being as distinct from overall psychosocial well-being. This phraseology will be used in this paper because it reflects the terminology of World Vision whose work is taken as the primary data source for this paper and because it is widely used in the increasing literature around faith and health. ‘Spiritual care’ will therefore denote a particular type of care ‘…that addresses and seeks to meet existential and spiritual needs and challenges in connection with illness and crisis’ (Hvidt et al. 2020). Spirituality therefore specifically addresses the need for meaning or purpose and a sense of connectedness (White 2000).

Finally, faith is often presented as the foundation of religion and spirituality (Fowler 1981, p.xiii). Religion and spirituality can be seen as functions of faith or value adds of faith (Newman 2004, p.106). The term faith is often seen as a more useful concept therefore to cover the wide range of beliefs, practices, structures and experiences that guide people's and communities’ lives. While faith is often understood as being experienced personally and privately, rooted in individual’s beliefs, it is central to the religious and spiritual expressions being studied here and in relation to humanitarian responses generally and MHPSS interventions in particular. Faith is therefore a general term that is foundational to, and inclusive of, the diverse range of religious beliefs and practices, spiritual care and lived experiences of communities of faith and their adherents.

Walker et al. prefer the more encompassing term ‘faith’ because it ‘best describes the hard-to-measure but central phenomenon that explains so much about why and how humans often prove resilience in the wake of hardships and disasters’ (Walker et al. 2012, p.118). They suggest that the term ‘faith’ is particularly relevant to the study of humanitarian crises and aid as it ‘refers to that which can sustain individuals in times of disaster and extreme hardship’ (Walker et al. 2012, p.118). Given the focus of this paper on MHPSS during and after humanitarian crises, the term ‘faith’, as per Walker et al.’s (2012) usage, will be used most widely in this paper. The term faith will be used in this paper to encompass the diverse range of religious and spiritual beliefs, practices and institutions and their lived expressions. If the terms ‘religion’ and ‘spirituality’ are used, it is in very specific instances when referring to their distinctive meanings and definitions and how they are experienced.

## Holistic support: a rights-based understanding of mental health and psychosocial needs in times of crisis

There has been a fair bit of critique about how global investment in mental health and MHPSS is low in relation to overall well-being (Mahomed 2020). Well-being is the focus of Sustainable Development Goal (SDG) 3, ‘Ensure healthy lives and promote well-being for all at all ages’, and most of the targets focus on physiological health, healthcare services. However, it is broadly understood that mental health, or psychosocial well-being, is an important component of well-being. ‘The absence of cures, and the dearth of preventive interventions… in part reflects a limited understanding of the brain and its molecular and cellular mechanisms. Where there are effective treatments, they are frequently not available to those in greatest need’ (Collins et al. 2011, p.27). Mental health services from a healthcare perspective, including medication and in/outpatient treatment for mental health illness, are costly, but an increasing body of evidence indicates that investment in this kind of mental healthcare is not the most effective use of resources. As a result, in recent years, there has been a shift in thinking from a biomedical model of mental health to one built around community approaches (Mahomed 2020, p.42). Mental health is increasingly seen as a question of holistic well-being, from a perspective considering social ecology, human rights and economics. This signals a shift to focus on preventive approaches more than treatment-oriented approaches, with research on mental health increasingly drawn to the role of building mental and social capital, community and systemic approaches to treating mental health concerns.

There has also been a growing call to view mental health from the perspective of human rights (see literature review in Mann et al. 2016), and in 2020, a team led by Peter Stastny introduced a framework to capture the critical elements in a rights-based approach to mental health crisis response (Florence 2020). First, such an approach would include a strong commitment to communication, dialogue and the presence with the person in crisis: ‘The reality or the belief that it is impossible to be heard and understood is often central to an individual’s mental health crisis’ (Stastny et al. 2020, p. 110). It also entails continuity of engagement with a person over time and flexibility to work with a person when and where they feel comfortable. Harm reduction in rights-based care entails destigmatising harmful acts and shame, offering people safe spaces of respite, and highly judicious use of medical treatment (Stastny et al. 2020, p.112-113). Finally, and importantly, mental health care that is rights-based addresses the holistic needs of people, ensuring their basic material needs are met, and that access to employment and economic stability is supported. ‘Many, if not most, crises manifested in emotional distress originate in interpersonal problems or environmental stressors. Such adversities can push someone from a state of adequate functioning to severe distress’ (Stastny et al. 2020, p.113). A human rights approach to MHPSS, therefore, would include mobilising a person’s social support networks, helping them to think of creative solutions to meeting their basic needs and, when necessary, providing them with material resources to meet those needs.

Proponents of a rights-based approach to mental health observe that researchers and leaders in neuroscience and mental health fields are themselves calling for ‘a multilayered and multisectoral approach to prevention and treatment... including... provision of living and working conditions that enable healthy psychosocial development, promotion of positive interactions within and between social groups, social protection for the poor, anti-discrimination laws and campaigns, and promotion of the rights of those with mental disorders’ (Mann et al. 2016, p.264). Such holistic rights-based approaches have been found to be more cost-effective, more sustainable through peer support and more culturally adaptable. Some studies have suggested that rights-based mental health programmes deliver results comparable to biomedical or treatment-based approaches and at a fraction of the cost (Mann et al. 2016, p.272).

Historically, the academic literature regarding the nature of mental health needs and care has focused on times of stability, not in contexts of crisis, war, disaster and other emergencies — though, interestingly in humanitarian and development circles, there has been more interest in psychosocial needs in humanitarian response than as a part of ongoing development initiatives. Over the last decade, increased research efforts have focused on the role of MHPSS in humanitarian responses (see Tol and van Ommeren 2012; Van Ommeren et al. 2015; Tol et al. 2020), corroborated by the significant engagement of the wider development and humanitarian spheres (e.g. Meyer 2013; Mukdarut et al. 2017; UNICEF 2018). Nonetheless, a recent global study called grand challenges in global mental health found common themes across contexts, and the need to prioritise ‘primary prevention of mental disorders… enable family and community environments that support mental health, understand adaptive and resilient responses to daily life stressors, and establish cross-national evidence on factors underlying mental health disparities’ (Collins 2020, p. 265). There is, therefore, reason to believe that our understanding of the impact of mental health crises affecting individuals and communities in times of stability may broadly apply in times of humanitarian crisis affecting entire communities or societies.

Exposure to war, violence and extreme natural events is a known risk factor for mental illnesses such as PTSD, depression and other emotional or behavioural problems. Crisis can produce or aggravate ‘profound adversities such as poverty, disempowerment, social exclusion, poor housing, gender-based and community violence, changes in family configuration and functioning, lack of social support, and discrimination/stigma, all of which are linked to mental health problems’ (Amone-P’Olak et al. 2014, p.430). Research on former child soldiers in Uganda suggested that other resulting mental health concerns brought less dramatic ‘diagnoses’, such as feelings of guilt and shame, disempowerment, a sense of loss of moral agency and general stress from living in a difficult situation (Amone-P’Olak et al. 2014, p.425). These concerns were attributed not only to the experience of the child soldiers during times of conflict but also to the social and economic milieu of living in a recovering society after a season of crisis.

The Inter-Agency Standing Committee, a United Nations humanitarian coordination body, published in 2007 a set of guidelines on mental health and psychosocial support in emergency settings. These guidelines are widely followed as offering an understanding of best practice in supporting mental health in times of humanitarian crisis. Rather than giving an explicit definition of MHPSS, these guidelines state that ‘Mental health and psychosocial problems in emergencies are highly interconnected, yet may be predominantly social or psychological in nature’ (IASC Guidelines 2007). Many different possible social and psychological problems which can affect mental health are listed, including those that were pre-existing prior to the breakout of a crisis, those induced by the crisis and those which could result from well-intentioned attempts to help affected communities. Some of the potential social problems include extreme poverty, discrimination, political oppression, family separation and social disruption and the breakdown of traditional support mechanisms. Psychological problems include those challenges often listed as mental health illnesses, such as alcohol abuse, depression and anxiety, trauma and grief.

In keeping with the human rights approach to mental health outlined above, the IASC guidelines use the image of a pyramid to demonstrate how people with a higher level of social resilience are likely to weather crisis more effectively. At the base of the pyramid are basic services and security which help support mental health for the majority of an affected population and directly above that community and family supports. Though focused non-specialised and specialised supports should be offered concurrently to the other types of support, they are at the tip of the pyramid indicating that they are needed by fewer people. The guidelines therefore advise that material or physical vulnerability factors should be addressed or at least referred by providers of psychosocial care. Recent efforts to develop MHPSS evidence-based interventions have also largely focused on social, communal and material elements of mental health while also including concerted efforts to offer appropriate treatment and specialised services to those suffering mental health illness.

The importance of mental health interventions that focus on removing barriers to well-being, especially in times of crisis, was recently highlighted by the UN Special Rapporteur on the right to health (Puras 2017) and has since been further emphasised in analyses of the COVID-19 pandemic, wherein ‘the public health challenges posed by the virus are mirrored by social isolation brought about by physical distancing and financial hardship brought about by economic inactivity. The mental health implications of these myriad concerns are not likely to be addressed through exclusively bio-medical interventions and will instead require a more holistic focus on well-being’ (Mahomed 2020, p.43). Therefore, the principles of rights-based mental health care are widely applicable to humanitarian MHPSS. We turn now to a discussion of spiritual support as a dimension of well-being and mental health in crisis situations.

## The roles of faith in holistic mental health

The emphasis on holistic responses within rights-based approaches to MHPSS creates the space in which to engage with faith. Faith is unavoidably and inextricably linked with the mental health and psychosocial well-being of individuals and communities for many people in all contexts. However, for those living in low-income and fragile contexts where the majority of people adhere to a faith tradition, it is even more difficult to disentangle faith from people’s and communities’ ability to cope with crises. It is therefore untenable to overlook the roles of faith for MHPSS during and following humanitarian crises.

Throughout history, local faith leaders and institutions such as churches and mosques, have cared for people with psychosocial concerns and mental illness (Schafer 2010, p.122). More recently, it is estimated that worldwide, 40% of people who experience mental health concerns continue to turn to clergy or churches as their first line of assistance (Schafer 2010, p.122). The roles that local faith leaders and communities play in MHPSS are situated more broadly in an understanding that they are often first responders to humanitarian crises, though there is limited research on the work that they do outside the scope of collaboration or dialogue with the international humanitarian community (Marshall 2004, p.897; Wisner 2010, p.129; Ager and Ager 2011, p.465; Fiddian-Qasmiyeh and Ager 2013, p.4). It is increasingly recognised that faith can play an important role in personal and community recovery from humanitarian crises. The literature broadly divides the importance of faith for responding to crisis into two areas: the first relates to intrinsic beliefs, ideas, narratives and worldviews, and the second is around extrinsic behaviours, rituals and practices (Ager et al. 2015, p.210).

Faith beliefs can help people and communities find meaning during and following crises (Walker et al. 2012, p.120-122; Clarke and Parris 2019, p.1). In fact, beliefs can be particularly powerful sources of meaning making (Bosworth et al. 2003). Beliefs can contribute to processes of meaning making which can give coherence to traumatic events, supporting psychological integration (Koening 2007) and helping to provide emotional comfort and reduce stress (Pargament and Cumming 2012, p.199). Beliefs can also enhance self-empowerment, helping people cope and find meaning in illness (Ebadi et al. 2009, p.345). Beliefs can contribute to an improved quality of life, reduced incidences of affective disorders, lowered rates of suicide and good relationships. They can increase giving and forgiveness fostering both individual and community resilience and they can encourage more optimistic worldviews that offer people a sense of meaning and purpose. Many find comfort after bereavement and loss from their belief in God or a higher power who is close to them (Schafer 2010, p.123).

Henderson et al.’s (2010) research shows that beliefs and coping mechanisms inspire positive adaptation through their transcendent, adaptive and transformational values. Following hurricane Katrina, faith promoted inner peace, self-esteem, perseverance and helping others, all of which contribute towards individual and community resilience (Henderson et al. 2010, p.297). Similarly, Ager et al.’s (2014) case study based in Ethiopia following the Eritrean border conflict highlights how people’s ability to cope in times of crisis is impacted by how they frame traumatic events. This case study demonstrates the influential nature of faith on how people perceive, explain and behave in reaction to traumatic events in a country that continues to suffer from man-made and natural disasters.

It is important to note that faith-based coping is not monolithic, and faith beliefs are lived, expressed and embodied differently within and across different traditions. In recognition of the scale of this diversity, Wisner (2010, p.30) argues that it is too large a task to accumulate detailed worldwide accounts of the diversity of people’s faith-based coping strategies and worldviews. He concludes that while it is important to engage with faith actors to use their teaching to help build resilience to natural disasters, it is not important for mental health providers to understand how theologians and lay people understand disasters (Wisner 2010, p.130).

Wisner’s reluctance to engage with faith beliefs, worldviews and imaginaries is symptomatic of the formal humanitarian’s historically notably secular framing of MHPSS interventions (Wisner 2010, p.130). The neglect of faith as dimensions of MHPSS programming in humanitarian contexts reflects broader humanitarian concerns that engaging with faith might compromise the humanitarian principles of impartiality and neutrality (Ager and Ager 2011). In reality, the marginalisation or active avoidance of faith in MHPSS programmes risks failing to connect with the agendas and capacities of faith communities (Ager et al. 2014, p.73). While MHPSS actors have frequently acknowledged the important role that faith plays in individuals’ and communities’ resilience to crisis, the avoidance of faith-based language, in favour of more technical vocabulary, marginalises faith actors (Ager et al. 2014, p.74). One of the repercussions of marginalising a core part of people’s experiences of disasters in the form of faith beliefs is that it ‘disconnects programming from the perceptions, agendas, and institutions of local faith communities’ (Ager et al. 2014, p.74).

Faith beliefs can foster positive and negative meaning-making processes with the potential to promote and hinder individual and community resilience. During the COVID-19 pandemic, stories have emerged, and World Vision national staff have recalled instances of faith leaders preaching that sin and disobedience to God are responsible for the virus.^1^ That being said, research shows that the effects of such beliefs on psychological aspects of resilience and recovery are unclear, and while certain beliefs can encourage fatalism or passivity and potentially undermine humanitarian programmes, this is not often the case. There is a distinction between beliefs and how they are lived and embodied. Even though a belief system might be replete with fatalism, when people are trapped in buildings, or stranded by floods, those same believers try to help (Wisner 2010, p.130).

Faith-based behaviours, rituals and practices can also be an important source of resilience in times of crisis. Faith practices have been shown to correlate with increased social support (Walker et al. 2012, p.122), ensuring assistance in times of stress and helping people find a way to cope and protect themselves against emotional disorders (Hill and Pargament 2003, p.69). The Ethiopian Orthodox practice of the mähebar, a socioreligious association of ten to thirty people who meet around once a month on the day of the saint they have chosen to honour, is an example of the benefits of local faith practices that promote mental health and psychosocial well-being (Pankhurst 1992, p.150; Ager et al. 2014, p.79). While the ceremony is significant to people’s faith, ‘the lay benefits are mainly expressed in terms of friendship and kinship’ (Pankhurst 1992, p.153). Members of a mähebar call each other brothers and sisters, and it provides a much-needed support network to its members. Local faith associations provide a basis for communality and mutual assistance and can be a potential means for pooling resources (Ager et al. 2014, p.79).

Faith practices that are deeply embedded in faith communities also help define passages through life (Fiddian-Qasmiyeh and Ager 2013, p.5). Faith practices can create a sense of normality and control over a stressful situation. The importance of practices at the local level is reinforced by the priority given to artefacts and spaces associated with faith in times of crises. Following disasters, prayer mats and the reconstruction of shrines, temples, synagogues, mosques and churches are seen as priority tasks (Fiddian-Qasmiyeh and Ager 2013, p.5). Prayer is another practice that can contribute to psychological resilience. In a survey carried out following the events of 9/11 in the USA, faith, including prayer and spiritual feelings, was the second most important way of coping, utilised by 90% of people (Peres et al. 2007, p.344).

That being said, certain faith practices may hinder resilience in ways that make it harder for individuals, families and communities to recover from the trauma of a humanitarian emergency. Following the 2004 Tsunami, in the Muslim majority province of Aceh Indonesia, people’s meagre savings in the form of gold and jewellery were swept away because they were kept at home to avoid using interest-based bank accounts prohibited by the Qur’an (Gaillard and Texier 2010, p.83).

There is a growing interest among humanitarians in the roles of faith, both beliefs and practices, for personal and community resilience to trauma in humanitarian contexts in academic and practice. This has led, for example, to the production of the IASC (2018) guidelines on *A faith sensitive approach in humanitarian response: guidance on mental health and psychosocial programming*, in collaboration with the Lutheran World Federation and Islamic Relief Worldwide, who had also previously partnered together to publish resources on *Developing Guidelines for faith-sensitive psychological programming* (Fitzgibbon and French 2017). This increased appreciation of the impact that faith has on individuals’ and communities’ mental health and well-being, and the accompanying guidance, has not yet incorporated widely into MHPSS programming, though (Ager and Ager 2015). Growing acknowledgement of the applicability of research on faith for MHPSS in the North and humanitarian contexts in the South (Walker et al. 2012, p.120) is particularly timely in the context of a pandemic which affected every country in the world and in which there was a renewed inquiry into the intersections between faith and mental health (Dein et al. 2020).

## Using the rights-based approach as a framework for measuring the challenges and opportunities for faith contributions to MHPSS

The above sections, and this one to follow, highlight how faith can provide opportunities and challenges for effective MHPSS for individuals and communities in response to humanitarian crises. The humanitarian imperative to do no harm, and the potential of both positive and negative impacts of engaging with faith in humanitarian settings, mandates humanitarian actors to approach faith collaborations for MHPSS with caution (Schafer 2010, p.125). In an internal survey of faith leader partners, all said that they are offering some form of psychosocial support to their congregants, and 60% said that they felt they had all of the skills they need to offer this kind of care. This raises some concern about faith leaders’ understanding of their own potential to do harm. An analysis of what faith leaders are doing within a framework of holistic care can therefore help better understand both the contribution made by faith communities and the support that they need to improve their contribution.

The human rights-based framework proposed by Stastny et al. (2020) briefly outlined above offers a useful set of types of care by which to analyse the data about what faith leaders are doing and able to do in supporting mental health, while also testing the relevance of this new framework to a real-life situation. By framing their response to the COVID-19 pandemic in these terms, the areas of contribution and the areas of risk for faith leaders playing a role in MHPSS begin to take a more clear shape. We see that local faith leaders provide MHPSS according to many of these types of care. While they may not have the medical or psychological expertise to provide some highly specialised medical and psychological treatment, they are able to provide MHPSS that extends beyond secular frameworks for MHPSS, contributing to a more holistic approach to MHPSS. This analysis can therefore help frame faith leaders’ role as MHPSS providers in their own right while exempting them from the work which would require medical or psychological expertise. For this analysis, the detailed framework offered by Stastny et al. (2020) has been simplified into six thematic areas of intervention.

### Communication, dialogue and the presence with the person in crisis

According to Stastny et al. (2020), the presence responds to the basic human need for companionship, a need which may be felt more acutely in times of crisis. ‘The reality or the belief that it is impossible to be heard and understood is often central to an individual’s mental health crisis. Connection to a trusted professional, friend, or “person with experience” can help resolve the immediate situation and avoid coercive consequences’ (Stastny et al. 2020, p.110). This may be the area in which local faith leaders most thrive. The above-mentioned survey refers to one-on-one counselling, praying with people and maintaining communication via phone or social media, as common activities cited by local faith leaders.

Support for congregations emerged in the sitrep analysis as a key theme across many different countries of World Vision engagement with faith partners. Online webinars for faith leaders covered themes such as ‘solidarity’, ‘gratitude’, ‘hope’ and ‘tenderness’. Staff reported that local faith leaders whom they know are regularly organising community and individual prayers. They used phone and online technologies and also in many countries visited people individually going door to door through the community.

Furthermore, the sitrep data highlights a commitment on the part of both World Vision and its faith partners to support congregations to facilitate the continuation of faith practices, rituals, rites and services during the COVID-19 pandemic. Activities included providing personal protection equipment (PPE) and WASH facilities in places of worship and developing guidelines on dignified funerals and other faith practices. Prayer was also prioritised. A number of World Vision’s national offices received prayer requests during the pandemic and engaged with local communities of faith online and through SMS to organise days and weeks of prayers.

Local faith leaders also supported one another to feel confident in their ability to continue to offer encouragement to members of their communities. In several countries, online support groups of faith leaders met regularly to encourage one another, recognising that each member spent most of their time in relationship with congregants. In one country, local faith leaders were invited to a session on ‘retreat and silence’, the description of which highlighted the need for faith leaders to self-care because of spending so much time with congregants facing uncertainty and fear.

### Continuity of engagement with a person over time

Continuity is often an elusive goal of mental health services, but there is widespread consensus that it is needed. ‘While some respond well to a one-time intervention, the offer of an ongoing relationship provides a powerful tool for persons in crisis to reconstitute their lives, even in the face of fractured connections’ (Stastny et al. 2020, p.112). Even if a service cannot continue for the long-term, Stastny et al. (2020) point out that at least continuity of one person or relationship would be good; they further observe that practices such as triage and referrals to service providers go against the principle of continuity. In humanitarian response, aid providers often are implementing short-term grants with a duration ranging from a few months to a year, which makes continuity of psychosocial care in humanitarian emergencies even more difficult than in times of stability.

Local faith leaders, if they are actively engaged in building community cohesion and supporting existing social networks, are well-placed to offer a degree of continuity. Where their care may be more relational than professional, local faith leaders are themselves members of a community, and therefore, any care they offer can be sustained over time. The sitrep data highlights numerous countries in which pre-existing networks were utilised by local faith leaders to continue offering support to their communities in the COVID-19 crisis; they rarely initiated new services or care but rather adapted to a socially distanced model of relating to people and in some cases increased their efforts to offer counselling, prayer or other types of support because they saw the emotional impact of the crisis on people in their networks. They also invested substantial effort into finding ways to continue meeting as a congregation or to resume meeting as soon as they could safely do so.

### Flexibility to work with a person when and where they feel comfortable and in a safe space

This feature of a rights-based approach to care is about ensuring that the person in distress feels most comfortable with the context in which care is offered. ‘Mobility, outreach and home visits recognising flexible location are key components of many community mental health services, including crisis intervention’ (Stastny et al. 2020, p.111). There was little in the data to support any premise that local faith leaders may seek to offer care to their communities in keeping with this principle, and it is likely that unless oriented to the importance of this flexible approach through appropriate training or dialogue with psychosocial care experts, some local faith leaders may be highly flexible and other local faith leaders fairly rigid in the times and places in which they would meet with congregants facing fear, uncertainty or grief — according to their personal and cultural styles. This is a risk of harm for local faith leaders who may not have been given an opportunity to reflect on their ability to offer flexibility as a form of care. Nonetheless, there were many mentions of local faith leaders conducting home visits when government guidance in their respective countries allowed them to do so, which is already a form of flexibility which many professional and humanitarian actors lack the resources to do.

Stastny et al. (2020) also emphasise the value of meaningful peer involvement in which peers share their personal experiences with others facing crisis. World Vision’s approach to mobilising local faith leaders adopts a peer support model, by encouraging local faith leaders to identify key members of their congregations to invite to training and then meet regularly in order to collaborate for meeting material or social needs. Therefore, many of the faith communities with which World Vision has had pre-existing programming had networks in place prepared to connect peers and empower supportive networks within congregations, many of which proved to be highly flexible to the sudden needs sparked by the pandemic, adaptable to personal needs and connections. On the flipside, though, there are many contexts in which faith leaders are known as authorities with some social distance from their congregants, which would make it difficult for this kind of potentially informal support to thrive.

### Destigmatising harmful acts and shame

Closely linked to harm reduction, and the overall principle of do no harm, a rights-based approach to mental health care will encourage reduction in harmful acts without stigmatising such behaviour. The literature on this topic primarily is concerned with substance abuse, self-harm and other risky behaviours. However, it may apply in the context of a humanitarian health crisis such as that brought on by COVID-19, because health and hygiene behaviours are either promoted or stigmatised. The focus of this destigmatising approach would be to maintain ‘a collaborative stance with the person, who may be ashamed and fearful of losing rights due to such behavior, when seeking help’ (Stastny et al. 2020, p. 113).

There is extensive evidence of faith leaders stepping up in promoting positive health and hygiene practices. Faith leaders emerged in the World Vision data as conduits of government health messaging and global best practices to their community. In the survey of faith leaders, 76% of respondents said that the most important things for members of their communities to do in the pandemic were social/physical distancing, hand washing and wearing masks. They also reported a high level of reliance on government health ministries for getting updated information and stated that they are actively teaching and preaching about health practices both from the pulpit and in informal conversations. World Vision played an active role in mobilising faith leaders to play this role, through sending them updated information and conducting brief trainings on COVID-19 public health principles.

At the same time, faith leaders were encouraged to counter stigmatisation of groups which have been blamed for the spread of the virus. In many countries, the sitrep data referred to activities designed to support faith leaders’ efforts to debunk rumours about the virus and resulting social stigmas. Key informants stressed false information, and rumours of COVID-19 being a hoax were significant obstacles to tackling COVID-19. There was a concerted effort on the part of many local faith communities to correct misinformation. However, we also must acknowledge that there are likely contexts in which faith communities not receiving information and support from a humanitarian organisation such as World Vision could be themselves key actors in spreading misinformation.

What is unclear, however, from the data available, is to what extent faith leaders were potentially stigmatising or destigmatising those engaged in health and hygiene practices which are not recommended best practice according to the public health community. There is little evidence to reassure us that faith leaders were not inadvertently contributing to the stigmatisation of community members who are ignoring health guidance.

### Highly judicious use of medical treatment

This principle emphasises the importance of seeing psychotropic medications as a last resort, but not ruling it out entirely. The data suggests that faith leaders are rarely connected to psychological or medical healthcare providers, although they can play an important role in referring people to psychological or medical professionals. Similarly, unless they are dual career, there is no evidence that they would or should be playing a role in dispensing medication for psychosocial purposes.

### Response to basic needs

Stastny et al. (2020) write that a gap in basic needs related to access to nutrition, clothing, funds, housing, etc. can trigger or deepen distress. Therefore, psychosocial support which responds to basic needs ‘may involve mobilizing a person’s natural support system, collaborating with him or her on problem-solving, and even providing material resources, such as food, clothing, or money, which will yield desired results quickly’ (Stastny et al. 2020, p.113-114). This is certainly an area in which faith leaders are particularly active.

There were numerous examples given in the sitrep data of local faith leaders collecting food and arranging for it to be distributed to vulnerable families in their communities or identifying the most needy households to which humanitarian organisations such as World Vision could distribute food or hygiene packs. There was also frequent mention of local faith leaders themselves going without in order to help more needy families, then efforts to meet the basic needs of faith leaders many of whom themselves suffered the economic fall-out of lost income due to cancelled religious services. For many faith communities, caring for body and soul was described as part and parcel. One example which illustrated this was of a psychological first aid training for faith leaders which became the impetus for arranging a distribution of masks. Unlike in some of the other aspects of care above, the sitrep data outlines various examples in which faith leaders themselves took initiative, without encouragement or nudging from World Vision, to identify and meet basic needs and to couple that support with social or faith-based activities.

There are some common concerns about faith communities’ potential to do harm when responding to the fall-out of crises. Concerns are often raised about the inclusion of evangelism or proselytism in faith-inspired MHPSS responses (Schafer 2011, p.75, 122), which would threaten the humanitarian principles of neutrality and impartiality. Another concern relates to the potential exclusion, marginalisation or essentialisation of women, girls (Bradley 2011, p.26-28) and other groups including lesbian, gay, transgender, queer and intersex plus (LGBTQ+) communities. There is limited ad hoc evidence around whether, or in what circumstances, faith communities’ involvement in MHPSS realises these concerns (Ager et al. 2015, p.465). Nevertheless, it is clear that suitably sensitive and faith literate dialogue needs to accompany the inclusion of faith in MHPSS, to ensure that faith communities’ engagement does no harm (Onyango 2011, p.64; Schafer 2011, p.74; El Nakib and Ager 2014).

Faith is not a magic bullet to improve mental health and psychosocial well-being following stressful events. There are opportunities and challenges to engaging with faith for a rights-based approach to MHPSS in humanitarian contexts. The COVID-19 pandemic highlighted the diverse ways that faith was being engaged with to respond to the pandemic and its secondary impacts, including MHPSS, in emergency settings. It is clear therefore that faith is an important consideration for holistic approaches to MHPSS in humanitarian contexts.

## Spiritual support as an end in itself

As we have argued thus far, there is an increasing appreciation of the potential role that faith-based beliefs and practices can play as part of a holistic approach to promoting a secular framing of MHPSS. However, discussions around the roles of faith in MHPSS continue to overlook, or actively avoid, engaging with spiritual well-being as an end in itself. This potentially overlooks part of faith communities’ distinctive offering to MHPSS. Following traumatic events, having faith-based beliefs can cause some additional stress but may also contribute to better adjustment as the traumatic events are integrated into a new meaning system (Ai and Park 2005, p.246). As a result, faith can be a distinctive source of recovery from trauma in this respect (Matthews and Marwit 2006).

While faith-based beliefs and practices can help people manage stressful situations, if people’s faith in those beliefs and practices are shaken, that can cause additional pain and suffering to those impacted by humanitarian crises. Faith-based struggles can mark a kind of second trauma, causing additional stress and anxiety that negatively impact mental health and psychosocial well-being. If not addressed, these faith-based struggles can lead to further harm. The secondary trauma of potentially losing or questioning one’s faith may require specialised psycho-spiritual support (Clarke and Parris 2019, P.5).

Pargament et al. (1998) identify two patterns of faith-based coping. The positive pattern includes forgiveness, seeking spiritual support, collaborative faith-based coping, faith connection, purification, and benevolent faith-based reappraisal. The negative pattern consists of spiritual discontent, punishing God reappraisals, interpersonal faith-based discontent, demonic reappraisal, and reappraisal of God’s powers. In a study by Wortmann and Park (2009), they suggested that while many found meaning and acceptance of their losses in experiences and practices of faith, an equivalent number of people saw their losses contribute to significant struggles including questioning or losing faith.

Disaster can change perceptions of the divine and challenge previously held and accepted worldviews (Schafer 2011, p.74). The literature on ‘Belief in a Just World’ (BJW) (Wenzel et al. 2017) highlights that people with faith-based beliefs tend to see the world as a just place, and that good people are rewarded and bad people are punished. There is evidence, albeit limited, that these findings are similar in humanitarian contexts (Clarke and Parris 2019, P.5). Clarke and Parris’s (2019, p.5) interviews with subjects who had experienced a variety of humanitarian events — natural and man-made — experienced those events through the lens of faith. Respondents detailed that viewing these experiences through a faith lens led to positive and negative worldviews depending on the individual. However, trauma involving loss and devastation can cause established worldviews to crumble, confronting people with the reality of a world in which people suffer unfairly. Janoff-Bulman (1992) suggested that trauma shatters our assumptions about the world. As a result, recovery from trauma involves building new assumptions and a worldview that incorporates the traumatic experiences.

Therefore, affected communities in crisis often express a deep need for spiritual support activities such as faith-based counselling, prayer, spiritual guidance, peer support, opportunities to explore issues of faith, participating in faith practices and facilitating rituals (Paratharayil 2005; Schafer 2010, p.125; Fiddian-Qasmiyeh and Ager 2013, p.35). Therapeutic narrative is often used in local faith communities to make connections within the wider community, encouraging feelings of hope (Fiddian-Qasmiyeh and Ager 2013, p.36).

The sitrep data from World Vision’s F&D responses to COVID-19 supports our argument that individuals and communities, including in humanitarian contexts, seek spiritual support during times of crises, which is often provided by faith actors. The sitrep data gives numerous examples of local faith leaders finding creative ways to provide spiritual support during the COVID-19 pandemic, including reaching members of the community through spiritual group counselling and providing spiritual counselling online or through apps. There are also examples of spiritual support being offered as a child protection strategy, providing virtual prayers, sunday school and other activities, not only to support children’s spiritual development nurture but also to ensure their social and emotional needs are met. Many faith leaders partnering with World Vision mentioned that they saw the most urgent needs of their communities to be for hope, a sense of meaning and encouragement.

The sitrep data also highlights that while local faith leaders played a vital role in supporting the most vulnerable, faith leaders themselves are highly vulnerable during crises. Many lost their income due to cancelled gatherings. In some cases, they themselves bore the brunt of the economic fall-out and poverty. Many local faith leaders also hold a degree of responsibility for the well-being of those around them so may feel overwhelmed and under equipped. As a result, many local faith leaders and congregations received targeted support to continue providing spiritual and pastoral support to their communities as part of their holistic response to the pandemic and its secondary impacts.

The sitrep data shows that it is often hard to distinguish local faith leaders’ MHPSS activities, which include spiritual support, from local faith leaders’ calling more broadly to shepherd their congregations. This may be because, as the data shows, spiritual support is widely seen as part and parcel with MHPSS. For many faith actors, spiritual support is integrated into their broader responses to crises. For example, the sitrep data gives examples of faith leaders conducting home visits, when the national guidance allows it, to disseminate COVID-19 preventative measures, during which they are integrating their pastoral roles and counselling services. This may reflect the fact that for many people and communities of faith, spiritual well-being is integral to their psychosocial well-being. Or perhaps, they are integrating elements of MHPSS into their spiritual care — that is, mental health and psychosocial well-being are integral to their spiritual well-being.

Spiritual support for humanitarian responses not only is the remit of faith-inspired actors but also is an important consideration for all humanitarian actors. In their interviews with faith-inspired and secular humanitarian agencies, Clarke and Parris (2019) found that all agencies confirmed their ability to include the faith-based worldviews of affected communities in their programming. A Christian FIO like World Vision may be well positioned to provide spiritual nurture in times of crisis, evidenced by communities approaching World Vision to request spiritual support for local churches, communities and congregations. In Schafer’s (2010) experience of providing spiritual support as part of MHPSS interventions in Haiti, as a Christian organisation with clear faith values, who speak the same faith-based language, World Vision felt comfortable and competent to provide spiritual support (Schafer 2011, p.76). During the COVID-19 pandemic, it was predominantly FIOs who most rapidly and significantly integrated spiritual support into their responses.

That being said, IFIOs like World Vision have their own distinct challenges for providing spiritual support to all who need it. While they may be well placed to provide spiritual nurture to those of a Christian faith, faith actors including IFIOs may feel less comfortable providing for individuals, families and communities of other faiths (Schafer 2010, p.125). This is an important challenge for any FIO because to neglect the spiritual nurture of people of non-Christian faiths and beliefs may be seen as discriminatory. It is also important that IFIOs recognise the diversity of faith-based beliefs and practices in a given context to ensure compliance with the IDRC/ICRC Code of Conduct and people of all faiths have access to MHPSS programmes that are culturally, religiously and spiritually appropriate (Schafer 2010, p.122). World Vision’s sitrep data includes numerous examples of, rather than providing spiritual support itself, engaging with and supporting local faith leaders of all traditions. Outside of Christianity, the data suggests these activities most frequently engage with Muslim leaders in Muslim-majority countries, but it also mentions engaging with Buddhist and Hindu leadership, to offer spiritual support to their own flocks.

## Conclusion: who defines the goals?

While there is increased engagement with faith for MHPSS in emergency settings generally, engagement with spiritual support remains limited. While there is limited engagement with the roles of spiritual support for a holistic approach to MHPSS by the humanitarian sphere, data emerging from the COVID-19 pandemic shows that this does not mean that it is not happening. Communities of faith are finding creative ways to provide spiritual support to promote people’s ability to cope with the pandemic and accompanying lockdowns. It is difficult to speak to the scale of these activities because they are often integrated into faith actors’ broader responses to COVID-19. Nevertheless, it is clear that spiritual support is an integral part of many communities of faith’s responses to COVID-19 and inseparable from their broader interventions to support people’s mental health and psychosocial well-being. The COVID-19 pandemic underscores that MHPSS interventions need to take faith-inspired beliefs and practices, as well as the secondary trauma of faith-based struggles, seriously when acting to promote what is often an integrated sense of mental health, psychosocial and spiritual well-being. The literature on MHPSS is not surprisingly written from a perspective of the importance of good mental health, and the literature about faith presents it as an aspect or contributor to this. This reinforces the idea that psychosocial well-being is the goal, and spiritual well-being may be a conduit to the real goal. However, many faith traditions have their own literature and teachings wherein spiritual well-being is highlighted as the ultimate goal, and if good mental health helps bring one closer to God, that is seen as bonus. We conclude this article with a reflection on whether it matters which is seen as the goal of supportive care.

Local faith leaders who have a genuine concern for the well-being of their congregants described their concern for the impact of the COVID-19 crisis in a holistic way: they were concerned about lost income, health risks, missed education and the emotional fall-out. However, as local faith leaders, they repeatedly described their primary concern as the fear, uncertainty and lost hope which they saw many people facing. While committed to caring for the practical needs of their congregations, this seems like a natural expression of compassion, whereas many of them described their primary job as being to restore a sense of hope and meaning through their preaching and teaching and to pray with and encourage congregants.

The World Vision teams who work with faith leaders seem to take a wide diversity of approaches, which may be largely a function of geography. While this paper has explored this theme from the perspective of a global pandemic evoking similar issues around the world, there are certainly some geographical differences. While it is risky to generalise, in the Latin American countries, there was a much greater emphasis on collaborating with faith leaders to arrange sunday school alternatives for children, strengthen spiritual support groups and give faith leaders the skills to teach about topics such as hope in times of crisis and ensure that faith communities could stay connected through the pandemic. In comparison, in southern African countries, there was greater emphasis on instilling good health and hygiene practices and offering psychosocial support to congregations while ensuring basic needs were met. In East African countries, there was greater mention of faith leaders engaging in advocacy to ensure that the needs of faith communities and simultaneously the needs of children were addressed through policy. In some countries around the world, most likely due to whatever the government guidance was in those countries, there were concerted efforts to help congregations resume meeting in a COVID-secure manner; while this need was not mentioned in many other countries.

Therefore, there seems to be a cultural and contextual element to answering the question of what is or should be the goal of supportive care in humanitarian settings. However, local faith leaders placed a much greater emphasis on praying with people, finding ways to meet with them one-to-one for encouragement, ensuring that children had meaningful faith activities in which to engage, and preaching messages of hope and meaning than might be expected by humanitarians or mental health professionals. This has a few important implications. The first is to recall that while faith communities can be powerful providers of mental health care, especially from the lens of a rights-based approach to mental health, they are first and foremost brokers of spiritual care. Second, though, is to challenge the notion that people’s core needs are more for mental health or well-being, than for a sense of spiritual peace and confidence. Local faith leaders around the world are keenly aware of people’s material needs but remind us that those needs may not be more ‘basic’ than the need to be certain of one’s existential and transcendental position and the sense of well-being that comes from a feeling of being ‘right with God’.

## Data Availability

As stated on the manuscript, the data that support the findings of this study are available from World Vision International, but restrictions apply to the availability of these data, which were used under license for the current study, and so are not publicly available. Data are however available from the authors upon reasonable request and with permission of World Vision.

## Footnotes

1 See Pulse (2020) CAN president says sin and disobedience to God are responsible for coronavirus, [online], [date accessed: 04/10/2022], available from: https://www.pulse.ng/news/local/can-president-says-sin-disobedience-to-god-responsible-for-coronavirus/z1ljqtf
